# Self-Etching Ceramic Primer Affects Surface Topography and Roughness of Two Zirconia-Reinforced Lithium Silicate Ceramics

**DOI:** 10.1055/s-0044-1787283

**Published:** 2024-10-11

**Authors:** Feras Alhalabi

**Affiliations:** 1 Department of Conservative Dental Sciences, College of Dentistry, Prince Sattam Bin Abdul-Aziz University, Al-Kharj, Saudi Arabia

**Keywords:** self-etching ceramic primer, lithium meta-silicate ceramic, surface roughness

## Abstract

**Objective** This article evaluates the etching efficacy of a self-etching ceramic primer (SECP) on zirconia-reinforced lithium silicate (ZLS) ceramics.

**Materials and Methods** Celtra Duo (DeguDent GmbH, Hanau-Wolfgang, Germany) and Vita Suprinity (Vita Zahnfabrik, Bad Säckingen, Germany) were used in this study. A total of 36 ceramic slices were prepared from each ceramic material and randomly distributed into three groups according to the surface treatment applied ( *n* = 12 per group). Group 1 (polished) was polished with silicon carbide paper discs and did not undergo any surface treatment; group 2 (SECP) was surface treated with SECP (Monobond Etch and Prime, Ivoclar Vivadent, Schaan, Liechtenstein); group 3 (hydrofluoric acid [HF]) was surface treated with 4.7% HF etching. Half of the specimens ( *n* = 6) from each group were gold-sputtered, and the surface topographic alterations were evaluated by scanning electron microscopy at magnifications of 5,000× and 10,000 × . The surface roughness of the other half ( *n* = 6) from each group was tested using a three-dimensional optical profiler. Data were statistically analyzed using two-way analysis of variance and Tukey's multiple comparisons test.

**Results** Both SECP and HF etching surface treatments resulted in a statistically significant increase ( *p* < 0.05) in the surface roughness of both ceramic materials, compared to that of their respective control group specimens (polished). HF etching resulted in a significant dissolution of the glassy phase of each ceramic.

**Conclusion** SECP can effectively etch ZLS ceramics. The etching patterns created after the application of SECP were mild compared to those produced by HF etching. The topographic surface features of ceramics are affected by both, surface treatment and material composition.

## Introduction

The popularity of indirect restorations made with digital technology has increased among dental practitioners owing to their reliable long-term clinical performance due to their excellent mechanical and esthetic properties. 1 2 Such indirect restorations are usually fabricated from two categories of materials: dental ceramics and indirect composites. Dental ceramics can be classified into oxide ceramics, such as zirconium dioxide (zirconia) and glass-based ceramics. Glass-based ceramics are mainly composed of glass phase and crystalline phase. They are subdivided into four types: (1) feldspathic ceramics, (2) leucite-reinforced ceramics, (3) lithium disilicate ceramics, and (4) zirconia-reinforced lithium silicate (ZLS) ceramics that contain additional zirconia. 3

Regardless of the type of the glass-based ceramic, the bond strength of resin-ceramic is a determinant for the clinical performance of ceramic restorations such as nonretentive partial ceramic crown. 4 Based on the glass phase and crystalline phase content of glass-based ceramic materials, resin-ceramic bonding is a two-step procedure; the first step involves the hydrofluoric acid (HF) etching to dissolve the superficial glass phase in the ceramics, creating significant topographic changes to enhance micromechanical bonding. 3 5 The second step involves the application of a 3-methacryloxypropyltrimethoxysilane (silane)-based primer to enable chemical adhesion between the primed glass-ceramics and methacrylate-based materials such as resin-cements or adhesives. 3 6 Despite the reliable bond strength achieved by this multistep approach, there is a risk of technical errors occurring during HF application, deactivating, washing, and post-etch cleaning. For example, prolonged HF etching can deteriorate the mechanical properties of glass ceramics due to excessive dissolution of the glass phase, and inadequate post-etch cleaning can affect the resin-ceramic bond strength due to presence of residue on the ceramic surface after etching. 7 8 In addition, HF is a toxic and hazardous material that can induce immediate nasal inflammatory responses by inhalation. 9

Previously, some materials such as titanium tetrafluoride and acidulated phosphate fluoride were suggested as alternatives to HF for etching lithium disilicate and feldspathic ceramics; however, none of them possessed an efficacy comparable to that of HF. 10 11 Recently, a self-etching ceramic primer (SECP) has been proposed to combine the effects of HF etching and silane priming, and it offers a safe, less time-consuming, and less technique-sensitive surface treatment of glass-based ceramic materials. 12 13 SECP showed promising results in several *in* *vitro* studies that evaluated the effects of SECP on bonding of lithium disilicate glass ceramics using shear and microshear bond strength tests. 12 14 15 However, more studies are required to evaluate its etching efficacy on other types of ceramics, such as ZLS ceramic materials used for fabrication of full-coverage restorations such as crowns and partial coverage restorations such as onlays. ZLS ceramic materials can also be utilized to fabricate ceramic veneers. 5 Therefore, the objective of this study was to assess the etching efficacy of SECP on two ZLS ceramic materials. The null hypothesis is, there would be no difference between the surface roughness of ZLS obtained following the surface treatment with either HF or SECP.

## Materials and Methods

### Specimen Preparation

Two ZLS ceramics, Celtra Duo (DeguDent GmbH, Hanau-Wolfgang, Germany) and Vita Suprinity (Vita Zahnfabrik, Bad Säckingen, Germany), were used in this study ( Table 1 ). The blocks of each ceramic material were sectioned using a 4-inche diamond cutting blade (IsoMet Blade, Buehler, Lake Bluff, Illinois, United States) mounted on a low-speed precision cutting saw (IsoMet 1000 Linear Precision Saw, Buehler) into a total of 36 slices of approximately 2-mm thickness. The ceramic slices were polished under water coolant with #600 silicon carbide paper disks attached to a grinding machine (Automata, Jean Wirtz, Germany) performing 200 revolutions per minute. The polished ceramic slices were subjected to ultrasonic cleaning with distilled water for 10 minutes and air-dried for 30 seconds. The sintering of Vita Suprinity slices was performed according to the manufacturer's instructions using VITA V60 i-Line PLUS, Vita Zahnfabrik. 16

**Table 1:** The composition of the materials used in the study

Group (surface treatment)	Celtra Duo
Monobond Etch and Prime, Ivoclar Vivadent, Schaan, Liechtenstein	Ammonium polyflouride, trimethoxypropyl methacrylate, solvents (alcohol and water), food colorant (fast green)
Vita Suprinity, Vita Zahnfabrik, Bad Säckingen, Germany	SiO _2_ , Li _2_ O, K _2_ O, P _2_ O _5_ , Al _2_ O _3_ , ZrO _2_ , CeO _2_ , pigments
Celtra Duo, DeguDent GmbH, Hanau Wolfgang, Germany	Lithium silicate with ∼10% ZrO _2s_

### Surface Treatment

The specimens of each ceramic material were randomly distributed into three groups according to the surface treatment applied ( *n* = 12 per group). In group 1 (polished), no surface treatment was performed. In group 2 (SECP), Monobond Etch and Prime, Ivoclar Vivadent, Schaan, Liechtenstein, was applied with a microbrush onto the top surface of the ceramic specimens, agitated for 20 seconds with a slight pressure, and allowed to react with the ceramic for 40 seconds. Subsequently, SECP was thoroughly rinsed with water and air-dried for 10 seconds. Specimens of group 3 (HF) were subjected to 4.5% HF (IPS Ceramic Etching Gel Ivoclar Vivadent) etching. HF was applied with a small disposable brush onto the top surface of each ceramic specimen; the treatment duration was 20 seconds for Celtra Duo and 30 seconds for Vita Suprinity, according to the manufacturer's recommendations. The remaining HF was removed by intensive spraying with water, followed by air drying. The etched ceramic specimens were ultrasonically cleaned for 5 minutes to remove any residue.

### Surface Topography Evaluation

Half of the specimens ( *n* = 6) from each group were dehydrated in ascending concentrations of ethanol before gold sputtering using a sputter coater (fine coat ion sputter JFC-1100, JEOL Ltd., Tokyo, Japan) for 3 minutes at 30 mA. Subsequently, the gold-sputtered ceramic slices were fixed onto brass stubs, and the surface topographic alterations were evaluated using scanning electron microscopy (SEM) (JSM-6610LV, JEOL Ltd.) at a magnification of 5,000× and 10,000× at a working distance of 8 mm. SEM was operated at 20 kV.

### Surface Roughness Evaluation

The surface roughness (Ra) of the other half of the specimens ( *n* = 6) from each group was evaluated using a high-resolution three-dimensional (3D) noncontact optical profiler (Contour GT-K 3D Optical Microscope, Bruker, Billerica, Massachusetts, United States). The specimens were vertically scanned at ×5 Michelson magnification and a field of view of 1 × 1 mm. The scan speed was 1 × , and thresholding was 4. The software used for the analysis and graphical output was Vision 64 (Bruker, Billerica). Four scans were obtained and averaged for each specimen.

### Statistical Analysis

A two-way analysis of variance (ANOVA) test was utilized to examine the effect of “surface treatment” and “ceramic material” as well as their interactions on the obtained surface roughness (Ra). Tukey's multiple comparison test was used to evaluate the differences between the tested groups. The statistical analyses were performed using R software version 4.1.2, R Foundation for Statistical Computing, Vienna, Austria.

## Results

### Surface Topography

**Fig. 1:**
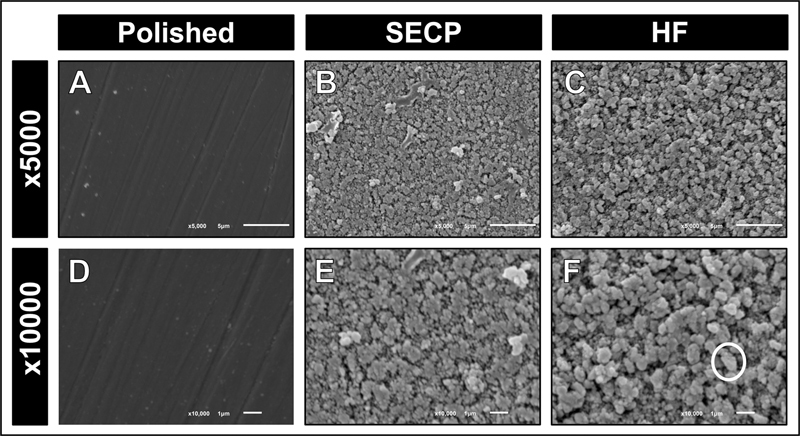
Representative scanning electron microscopy (SEM) photomicrographs at 5,000× and 10,000× of Celtra Duo after polishing ( **A** , **D** ) the smooth surface with no etching pattern; self-etching ceramic primer (SECP) surface treatment ( **B** , **E** ) with a mild etching pattern; hydrofluoric acid (HF) etching ( **C** , **F** ) with an aggressive etching pattern. White circle: metasilicate crystals are larger in size compared to those in Vita Suprinity ( Fig. 2F ).

**Fig. 2:**
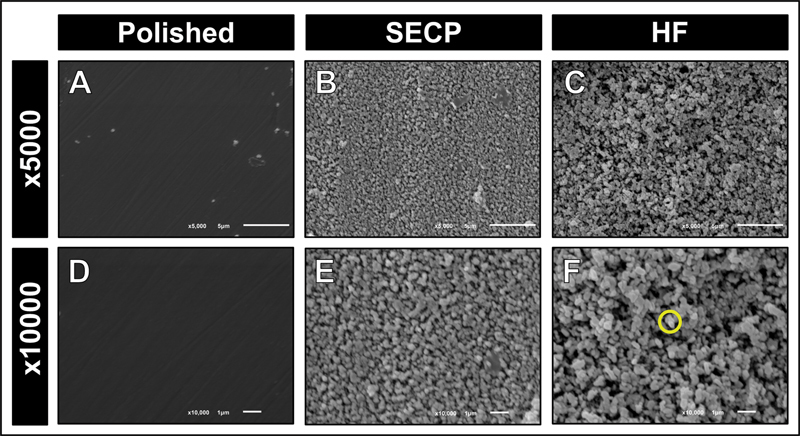
Representative scanning electron microscopy (SEM) photomicrographs of Vita Suprinity at 5,000× and 10,000 × . Polished ( **A** , **D** ) smooth surface with no etching pattern; self-etching ceramic primer (SECP) surface treatment ( **B** , **E** ) with a mild etching pattern; hydrofluoric acid (HF) etching ( **C** , **F** ) with an aggressive etching pattern. Yellow circle: metasilicate crystals are smaller in size compared to those in Celtra Duo ( Fig. 1F ).

Both lithium metasilicate and lithium orthophosphate crystals observed after HF etching were prominent. In contrast, SECP resulted in milder etching patterns ( Figs. 1B, E and 2B, E ). Surface microirregularities were less prominent than those created after HF etching; however, lithium metasilicate and lithium orthophosphate crystals were observed. The etching patterns obtained after HF etching or SECP application were markedly different. The etching of both ceramics with either 4.7% HF or SECP resulted in clear topographic surface alterations compared to the control group, in which a smoother and more homogenous surface topography was observed ( Figs. 1A, D and 2A, D ). HF etching resulted in an aggressive etching pattern with deeper and more numerous microporosities and grooves formed within the ceramic surface.

### Surface Roughness

**Fig. 3:**
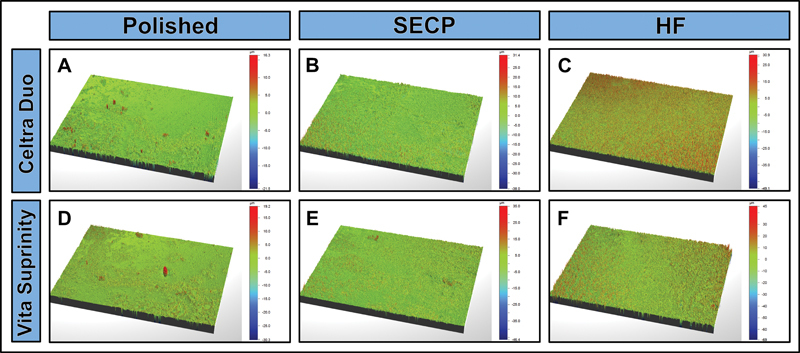
Three-dimensional representative optical profilometer images of the two ceramic materials (Celtra Duo and Vita Suprinity). Self-etching ceramic primer (SECP)-treated group specimens ( **B** , **E** ) presented significantly higher surface roughness compared to the polished (control) group ( **A** , **D** ). Hydrofluoric acid (HF)-treated group specimens ( **C** , **F** ) presented the highest surface roughness for both ceramic materials.

The mean and standard deviation surface roughness values for the tested groups are presented in Table 2 . Two-way ANOVA results ( Table 3 ) indicated that only the surface treatment had a significant effect on the obtained surface roughness ( *p* < 0.001). Both SECP and HF etching surface treatments resulted in a statistically significant increase in the surface roughness of both ceramic materials, compared to that of their respective control group specimens (polished). However, the surface roughness obtained after SECP surface treatment was significantly lower than that obtained after HF etching. Within each group (polished, SECP, and HF), there was no statistically significant difference between the surface roughness values of the two ceramic materials. Representative images for each group illustrate the effects of each surface treatment on the surface roughness of the ceramic. 3D representative optical profilometer images ( Fig. 3 ) illustrated the variable surface roughness of ceramics subjected to different surface treatments (polishing, SECP, or HF etching).

**Table 2:** Mean ± standard deviation surface roughness (Ra) expressed in (μm) of tested groups

Group (surface treatment)	Celtra Duo	Vita Suprinity
Polished (control)	1.021 ± 0.41 ^a^	0.95 ± 0.2 ^a^
SECP	2.28 ± 0.57 ^a^	2.3 ± 0.65 ^a^
HF	3.56 ± 0.79 ^a^	3.63 ± 1.11 ^a^

Abbreviations: HF, hydrofluoric acid; SECP, self-etching ceramic primer.

^a^ Indicate statistically significant difference.

**Table 3:** Two-way analysis of variance (ANOVA) results

Variable	Df	Sum Sq	Mean Sq	*F* -value	*p* -Value
Ceramic	1	0.01	0.000	0.000	0.882
Surface treatment	2	40.83	20.41	43.89	< 0.001 a
Ceramic * Surface treatment	2	0.03	0.015	0.031	0.941
Residuals	30	14.11	0.47		

Abbreviations: Df, degree of freedom; Sum Sq, sum squares; Mean Sq: mean squares.

a Indicates statistically significant effect.

## Discussion

The surface treatment of indirect ceramic restorations is an indispensable step to ensure the adequate cementation or repair of such restorations. This study was designed to evaluate the etching efficacy of SECP, as a new ceramic surface treatment, on two ZLS ceramic materials. This is clinically relevant because ZLS ceramic materials are extensively used in modern prosthetic dentistry while dental practitioners might be lacking essential information regarding the effect of novel surface treatments on their surface properties, particularly because there is no consensus on the use of SECP for surface treatment of ZLS ceramics. Both qualitative using SEM at different magnifications and quantitative using surface profilometer evaluations of the surface topographic features and surface roughness of ZLS ceramic materials following surface treatment with SECP or HF were considered. This is believed to provide a multiscale assessment of the surface changes. ZLS ceramic materials are composed of a glassy phase, crystalline phase, and small amount of tetragonal zirconia. 17 The crystallization of ZLS ceramic materials can be either full or partial (such as in Vita Suprinity), 18 which explains the need for sintering to such a ceramic material. The SEM examination of ceramic surfaces provides a comprehensive qualitative detailed evaluation of the surface topographic features through high-resolution micrographs at a high magnification 19 20 21 ; however, the use of a surface profilometer can provide a quantitative evaluation of the surface roughness; 22 thus, statistical analysis can be performed.

There was a statistically significant difference between the surface roughness values of the two ceramic materials following the surface treatment with SECP or HF. Therefore, the null hypothesis was rejected. This is in accordance with the SEM evaluation, in which milder etching patterns and fewer surface topographic changes were noticed in specimens after SECP, compared with HF etching. The marked difference between the respective etching patterns and surface roughness of SECP-treated and HF-treated ceramic materials can be attributed to the extent of reaction with the glassy phase in the ceramic materials, rather than the acidity, despite the different pH of each treatment. HF etching is the most reliable surface treatment of glass-based ceramic materials, such as ZLS. 23 HF etching depends on the chemical reaction between the silicon content in the ceramic materials and fluoride ions of the HF, which results in the dissolution of the glass content in the ceramic material. 3 Thus, a significant surface alteration is produced. The application protocol (time) depends on the composition (glass content) of dental ceramics. 23

The SECP etching effect is mainly related to its tetrabutylammonium dihydrogen trifluoride content, which can chemically etch silica-based materials. 24 According to the manufacturer's instructions, SECP should be rubbed onto the ceramic surface for at least 20 seconds to ensure an intimate contact between the SECP and the ceramic surface. Recent studies showed that prolonged application or active application mode (scrubbing) results in more dissolution of the ceramic's glass content, which creates more significant surface topographic alterations, 14 25 even though they are still less distinct compared to those produced by HF etching.

The surface topographic features of both ceramics were similar when they were subjected to the same surface treatment (HF or SECP). ZLS ceramics are composed of a zirconia-reinforced matrix phase, in which the major crystalline phase is composed of lithium metasilicate crystals. 18 26 27 However, in Celtra Duo, the lithium metasilicate crystals are larger in size compared to those in Vita Suprinity 27 and can provide a plausible explanation for the different surface topographic features observed in both ceramics following HF etching or SECP application. However, two-way ANOVA results showed the effects of the ceramic material on the obtained surface roughness. This can be explained by their similar glass content. One of the limitations of this study is that the surface treatments (polishing, SECP, and HF) were applied to a flat surface specimen, which may be less clinically relevant, as ceramic dental restorations usually have complex geometry, rather than a flat surface.

## Conclusion

The SECP can effectively modify the surface properties of both ZLS ceramic materials, resulting in a significant increase in the surface roughness of the ceramic. The etching patterns created after the application of SECP were less aggressive than those produced by HF etching. The surface topographic features of ZLS ceramic materials can be affected by both the nature of surface treatment and the composition of the ceramic.

## References

[1] Spitznagel F A Boldt J Gierthmuehlen P C CAD/CAM ceramic restorative materials for natural teeth J Dent Res 2018 97 10 1082 1091 29906206 10.1177/0022034518779759

[2] Zarone F Di Mauro M I Ausiello P Ruggiero G Sorrentino R Current status on lithium disilicate and zirconia: a narrative review BMC Oral Health 2019 19 01 134 31272441 10.1186/s12903-019-0838-x PMC6610968

[3] Tian T Tsoi J K-H Matinlinna J P Burrow M F Aspects of bonding between resin luting cements and glass ceramic materials Dent Mater 2014 30 07 e147 e162 24612840 10.1016/j.dental.2014.01.017

[4] Politano G Van Meerbeek B Peumans M Nonretentive bonded ceramic partial crowns: concept and simplified protocol for long-lasting dental restorations J Adhes Dent 2018 20 06 495 510 30564796 10.3290/j.jad.a41630

[5] Awad M M Alqahtani H Al-Mudahi A Murayshed M S Alrahlah A Bhandi S H Adhesive bonding to computer-aided design/ computer-aided manufacturing esthetic dental materials: an overview J Contemp Dent Pract 2017 18 07 622 626 28713119 10.5005/jp-journals-10024-2095

[6] Matinlinna J P Lassila L V Özcan M Yli-Urpo A Vallittu P K An introduction to silanes and their clinical applications in dentistry Int J Prosthodont 2004 17 02 155 164 15119865

[7] Hooshmand T Parvizi S Keshvad A Effect of surface acid etching on the biaxial flexural strength of two hot-pressed glass ceramics J Prosthodont 2008 17 05 415 419 18482364 10.1111/j.1532-849X.2008.00319.x

[8] Belli R Guimarães J C Filho A M Vieira L C Post-etching cleaning and resin/ceramic bonding: microtensile bond strength and EDX analysis J Adhes Dent 2010 12 04 295 303 20157658 10.3290/j.jad.a17709

[9] Lund K Refsnes M Ramis I Human exposure to hydrogen fluoride induces acute neutrophilic, eicosanoid, and antioxidant changes in nasal lavage fluid Inhal Toxicol 2002 14 02 119 132 12122575 10.1080/089583701753403944

[10] Klosa K Boesch I Kern M Long-term bond of glass ceramic and resin cement: evaluation of titanium tetrafluoride as an alternative etching agent for lithium disilicate ceramics J Adhes Dent 2013 15 04 377 383 23534032 10.3290/j.jad.a29381

[11] Kukiattrakoon B Thammasitboon K Optimal acidulated phosphate fluoride gel etching time for surface treatment of feldspathic porcelain: on shear bond strength to resin composite Eur J Dent 2012 6 01 63 69 22229009 PMC3252805

[12] Alrahlah A Awad M M Vohra F Effect of self etching ceramic primer and universal adhesive on bond strength of lithium disilicate ceramic J Adhes Sci Technol 2017 31 2611 2619

[13] Wille S Lehmann F Kern M Durability of resin bonding to lithium disilicate and zirconia ceramic using a self-etching primer J Adhes Dent 2017 19 06 491 496 29234754 10.3290/j.jad.a39545

[14] Dapieve K S Aragonez G C Prochnow C Different etching times of a one-step ceramic primer: effect on the resin bond strength durability to a CAD/CAM lithium-disilicate glass-ceramic J Adhes Dent 2021 23 02 133 143 33825427 10.3290/j.jad.b1079573

[15] Dimitriadi M Zinelis S Zafiropoulou M Silikas N Eliades G Self-etch silane primer: reactivity and bonding with a lithium disilicate ceramic Materials (Basel) 2020 13 03 641 32023979 10.3390/ma13030641 PMC7040894

[16] Vita Suprinity working instructionsAccessed may 16, 2024 at:https://mam.vita-zahnfabrik.com/portal/ecms_mdb_download.php?id=82430&sprache=en&fallback=&cls_session_id=&neuste_version=1

[17] Zarone F Ruggiero G Leone R Breschi L Leuci S Sorrentino R Zirconia-reinforced lithium silicate (ZLS) mechanical and biological properties: a literature review J Dent 2021 109 103661 33864886 10.1016/j.jdent.2021.103661

[18] Silva L HD Lima E Miranda R BP Favero S S Lohbauer U Cesar P F Dental ceramics: a review of new materials and processing methods Braz Oral Res 2017 31 01 e58 28902238 10.1590/1807-3107BOR-2017.vol31.0058

[19] Oliveira-Junior O B Buso L Fujiy F H Influence of polishing procedures on the surface roughness of dental ceramics made by different techniques Gen Dent 2013 61 01 e4 e8 23302371

[20] Moravej-Salehi E Moravej-Salehi E Valian A Surface topography and bond strengths of feldspathic porcelain prepared using various sandblasting pressures J Investig Clin Dent 2016 7 04 347 354 10.1111/jicd.12171 26088205

[21] Valian A Moravej-Salehi E Surface treatment of feldspathic porcelain: scanning electron microscopy analysis J Adv Prosthodont 2014 6 05 387 394 25352961 10.4047/jap.2014.6.5.387 PMC4211055

[22] Karayazgan B Atay A Saracli M A Gunay Y Evaluation of Candida albicans formation on feldspathic porcelain subjected to four surface treatment methods Dent Mater J 2010 29 02 147 153 20379024 10.4012/dmj.2009-016

[23] Bajraktarova-Valjakova E Grozdanov A Guguvcevski L Acid etching as surface treatment method for luting of glass-ceramic restorations, part 1: acids, application protocol and etching effectiveness Open Access Maced J Med Sci 2018 6 03 568 573 29610622 10.3889/oamjms.2018.147 PMC5874387

[24] Pande A Levitin G Mui D S Design of a novel wet-etch reactor and etch chemistries: simulations and experimental verification ECS Trans 2019 28 109 118

[25] Cardenas A FM Quintero-Calderon A S Siqueira F SF Do different application modes improve the bonding performance of self-etching ceramic primer to lithium disilicate and feldspathic ceramics? J Adhes Dent 2019 21 04 319 327 31432046 10.3290/j.jad.a42929

[26] Elsaka S E Elnaghy A M Mechanical properties of zirconia reinforced lithium silicate glass-ceramic Dent Mater 2016 32 07 908 914 27087687 10.1016/j.dental.2016.03.013

[27] Belli R Wendler M de Ligny D Chairside CAD/CAM materials. Part 1: measurement of elastic constants and microstructural characterization Dent Mater 2017 33 01 84 98 27890354 10.1016/j.dental.2016.10.009

